# A33 ENDOSCOPIC ULTRASOUND-GUIDED GASTROJEJUNOSTOMY VERSUS SURGICAL GASTROJEJUNOSTOMY AND ENTERAL STENTING FOR THE TREATMENT OF MALIGNANT GASTRIC OUTLET OBSTRUCTION: A SYSTEMATIC REVIEW AND META-ANALYSIS

**DOI:** 10.1093/jcag/gwab049.032

**Published:** 2022-02-21

**Authors:** J Benchaya, Y Chen, M Martel, A N Barkun, J Wyse, L Ferri, C S Miller

**Affiliations:** 1 Medicine, McGill University Faculty of Medicine and Health Sciences, Montreal, QC, Canada; 2 McGill University Health Centre, Montreal, QC, Canada; 4 Sir Mortimer B Davis Jewish General Hospital, Montreal, QC, Canada

## Abstract

**Background:**

Gastric outlet obstruction (GOO), often encountered in advanced malignancy, is associated with debilitating symptoms and decreased quality of life. Traditional management of this condition has been surgical gastrojejunostomy (SGJ) or enteral stenting (ES). While SGJ is highly effective, it is invasive and associated with high rates of morbidity. ES provides a less invasive approach with a lower risk of adverse events; however, it is associated with a significant risk of stent dysfunction with increased need for reintervention. Endoscopic ultrasound-guided gastrojejunostomy (EUS-GJ) is a novel modality in the management of GOO that aims to endoscopically bypass the obstruction with a lumen-apposing metal stent, with early studies suggesting good effectiveness and safety outcomes; but the data are limited.

**Aims:**

To perform a systematic review and meta-analysis comparing the clinical outcomes of EUS-GJ to more traditional treatments of malignant GOO.

**Methods:**

The study protocol was prospectively registered with the PROSPERO international database. The literature was systematically searched using MEDLINE, EMBASE and Web of Knowledge databases from inception through May 2021. Studies comparing EUS-GJ to ES or SGJ in patients with malignant GOO were included. Meta-analysis was performed with results reported as odds ratios (ORs) with 95% confidence intervals (CIs) using random effects models. The two primary outcomes of interest were clinical success without GOO recurrence and adverse events. Secondary outcome was technical success.

**Results:**

Ten studies with a total of 1016 patients were included. EUS-GJ was associated with higher clinical success without GOO recurrence compared to SGJ or ES [OR: 2.19, 95% CI: 1.18–4.09, heterogeneity: P = 0.10; I^2^ = 59%]. Subgroup analysis showed higher clinical success without GOO recurrence compared to ES [OR: 5.31, 95% CI: 3.07–9.17], but no significant difference compared to SGJ [OR: 1.69, 95% CI: 0.76–3.72]. EUS-GJ was associated with fewer adverse events compared to SGJ and ES [OR: 0.28, 95% CI: 0.14–0.55] and compared to SGJ alone [OR: 0.20, 95% CI: 0.10–0.37], but no difference was noted when compared to ES alone [OR: 0.53, 95% CI: 0.15–1.87]. EUS-GJ was associated with decreased technical success compared to SGJ and ES [OR: 0.26, 95% CI: 0.09 – 0.75] and SGJ alone [OR: 0.14, 95% CI: 0.04–0.48]; however, there was no difference when compared to ES alone [OR: 0.43, 95% CI: 0.05–3.44].

**Conclusions:**

EUS-GJ provides a robust bypass with lower risk of recurrent obstruction compared to ES and fewer adverse events compared to SGJ. High quality prospective studies are needed to further characterize the role of EUS-GJ in the management of malignant GOO.

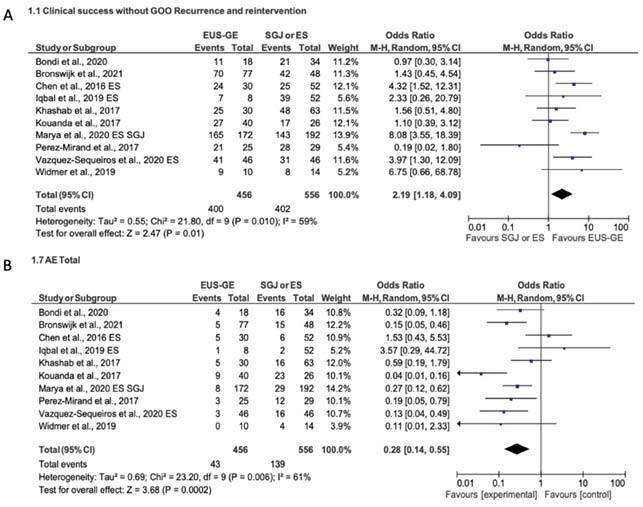

**Funding Agencies:**

None

